# Factors influencing therapy choice and clinical outcome in cerebral venous sinus thrombosis

**DOI:** 10.1038/s41598-020-78434-8

**Published:** 2020-12-10

**Authors:** Dagmar Krajíčková, Jiří Král, Roman Herzig, Ľudovít Klzo, Antonín Krajina, Jaroslav Havelka, Libor Šimůnek, Oldřich Vyšata, Tran Van Quang, Michal Bar, Martin Vališ

**Affiliations:** 1 Department of Neurology, Comprehensive Stroke Center, Charles University Faculty of Medicine and University Hospital, 500 05 Hradec Králové, Czech Republic; 2 Department of Neurology, University Hospital Ostrava and University of Ostrava Faculty of Medicine, 708 52, Ostrava, Czech Republic; 3 Department of Neurology, St. Anne’s University Hospital and Medical Faculty of Masaryk University, 656 91, Brno, Czech Republic; 4 Department of Radiology, Comprehensive Stroke Center, Charles University Faculty of Medicine and University Hospital, 500 05 Hradec Králové, Czech Republic; 5 Department of Radiology, University Hospital Ostrava and University of Ostrava Faculty of Medicine, 708 52, Ostrava, Czech Republic; 6 Department of Mathematics, Faculty of Nuclear Sciences and Physical Engineering, Czech Technical University, 160 00 Prague, Czech Republic

**Keywords:** Medical research, Neurology

## Abstract

We aimed was to assess the factors influencing therapy choice and clinical outcome after 3–4 months in patients with cerebral venous sinus thrombosis (CVST). In a retrospective, bi-centric study, the set consisted of 82 consecutive CVST patients (61 females; mean age 33.5 ± 15.7 years). Following data were collected: baseline characteristics, presence of gender-specific risk factors (GSRF), location and extent of venous sinus impairment, clinical presentation, type of treatment, recanalization, presence of parenchymal lesions, and clinical outcome after 3–4 months (assessed using the modified Rankin Scale [mRS], with excellent outcome defined as mRS 0–1). Multivariate logistic regression analysis was used for statistical evaluation. After 3–4 months, complete recovery was achieved in 41 (50%) and excellent clinical outcome in 67 (81.7%) patients. Female sex (OR 0.11; *p* = 0.0189) and presence of focal neurologic deficit (OR 0.16; *p* = 0.0165) were identified as significant independent negative predictors and, the presence of GSRF (OR 15.63; *p* = 0.0011) as significant independent positive predictor of excellent clinical outcome. In conclusion, in our CVST patients, the presence of GSRF was associated with excellent clinical outcome, while the female sex itself was associated with poorer clinical outcome.

## Introduction

The frequency of patients diagnosed with cerebral venous sinus thrombosis (CVST) has increased due to the expanded use of noninvasive sophisticated brain imaging methods, which enable to detect even clinically less presented or fully asymptomatic cases. Diagnosis based only on clinical data is difficult given the large variability of clinical symptoms. Besides the fact that CVST may present clinically with an isolated intracranial hypertension syndrome or nonspecific symptoms of encephalopathy, any potentially present focal symptoms do not respect the topographic patterns that are helpful for the diagnosis of arterial strokes. While according to a recent epidemiological study, CVST incidence in the whole population has been estimated as 1.31/100,000 persons, the incidence rises to 2.78/100,000 in women aged between 31 and 50 years^1^. The significantly more frequent incidence of CVST in young women can be explained by the presence of gender-specific risk factors (GSRFs), which include hormonal contraception (HC), pregnancy, puerperium, and hormone replacement therapy (HRT).

Although the prognosis of the disease is usually favorable, a poor outcome is seen in 5–13% of patients despite standard anticoagulation therapy^2–4^. In recent years, increased use of endovascular therapy can be seen in clinical practice; this therapy comprises two alternatives which can be applied either separately or in combination. Patients who are more likely to experience a worse outcome should be considered as the target group for this more aggressive therapeutic strategy. Nevertheless, conclusive evidence of the best treatment of CVST is not available.

We aimed was to assess the factors influencing therapy choice and clinical outcome (achievement of complete recovery and of excellent clinical outcome) after 3–4 months in patients with CVST.

## Results

In a retrospective, bi-centric study, the set consisted of 82 consecutive CVST patients (61 females; mean age 33.5 ± 15.7 years). Table 1 shows the baseline characteristics, including the presence of GSRF. Patients with GSRF were significantly younger than patients without GSRF (mean age 32.0 ± 11.1 vs. 42.0 ± 19.7 years; *p* = 0.0054). Other observed thrombophilic conditions included inherited thrombophilic conditions (factor V Leiden mutation, prothrombin mutation G20210A, JAK2 kinase mutation, MTHFR mutation and protein S deficiency), hematological disorders (myeloproliferation, anemia and lymphoma), Crohn’s disease, prostate cancer, craniocerebral trauma and smoking.

**Table 1:** Baseline characteristics of the groups according to the clinical outcome.

Modified Rankin Scale	0	0–1	2–6	*p* value (95% CI)
Number of patients (%)	41 (50%)	67 (81.7%)	15 (18.3%)	
Age, mean + SD (range); years	34.7 ± 17.0 (3–78)	33.0 ± 17.6 (3–78)	38.1 ± 15.1 (14–70)	0.316 (− 14.75–4.67)
GSRF, n (%)	28 (68.3%)	44 (65.7%)	5 (33.3%)	0.32
Deep vein thrombosis	1 (2.4%)	2 (3.0%)	0	1.0
**Clinical symptoms in the acute phase, n (%)**				
Impaired consciousness	5 (12.2%)	9 (13.4%)	5 (33.3%)	0.16
Focal neurologic deficit	14 (34.1%)	29 (43.3%)	12 (80.0%)	0.17
Neurologic deficit (NIHSS)	3.0 ± 7.0	2.1 ± 5.7	3.8 ± 6.3	0.31 (− 4.99–1.61)
**Parenchymal lesions on initial MRI, n (%)**				
Edema	15 (36.6%)	22 (32.8%)	7 (46.7%)	0.59
Hemorrhage	10 (24.4%)	15 (22.4%)	8 (53.3%)	0.15
Edema and hemorrhage	10 (24.4%)	14 (20.9%)	6 (40.0%)	0.35
**Impairment of sinuses**				
Extent (point score)	2.3 ± 1.6	2.7 ± 1.8	3.2 ± 1.7	0.37 (− 1.46–0.55)
Deep veins, n (%)	6 (14.6%)	15 (22.4%)	3 (20.0%)	1.0
Cortical veins, n (%)	7 (17.1%)	9 (19.1%)	2 (13.3%)	1.0

Values are expressed as n (%) or mean ± standard deviation. Fisher’s exact test was used for calculation of *p* values of categorical data and unpaired two-tailed t-test was used for calculation of *p* values of continuous data.

*CI* 95% confidence interval of the difference of the mean of group “0–1” minus group “2–6”, *GSRF* gender-specific risk factors, *MRI* magnetic resonance imaging, *NIHSS* National Institutes of Health Stroke Scale, *SD* standard deviation.

As presented in Table 2, anticoagulant therapy was used in 65 (79.3%) patients, intrasinus thrombolysis (IST) with administration of recombinant tissue plasminogen activator (rtPA) alone in 2 (2.4%), mechanical thrombectomy (MT) alone in 5 (6.1%) and in combination with IST in 10 (12.2%) patients. Endovascular therapy was used more frequently in women (24.6 vs. 9.5% of men) who generally more commonly suffered from impaired consciousness (18 vs. 14.3% of men), a focal deficit (52.5 vs. 38.1% of men) and a thrombosis of the deep cerebral venous system (27.9 vs. 14.3% of men).

**Table 2:** Treatment and achieved recanalization in the groups according to the clinical outcome.

Modified Rankin Scale	0	0–1	2–6	*p* value
Number of patients (%)	41 (50%)	67 (81.7%)	15 (18.3%)	
**Treatment, n (%)**				
Anticoagulant therapy	34 (82.9%)	54 (80.6%)	11 (73.3%)	1.0
Endovascular therapy				
IST alone	0	1 (1.5%)	1 (6.7%)	0.35
MT alone	2 (4.9%)	3 (4.5%)	2 (13.3%)	0.25
IST + MT	5 (12.2%)	9 (13.4%)	1 (6.7%)	1.0
**Recanalization after 3–4 months, n (%)**				
Complete	27 (65.9%)	37 (55.2%)	6 (40%)	0.62
Partial	11 (26.8%)	27 (40.3%)	6 (40%)	1.0
None	3 (7.3%)	3 (4.5%)	3 (20%)	0.10
Pathological finding on control MRI, n (%)	7 (17.1%)	15 (22.4%)	9 (60%)	0.06

Values are expressed as n (%). Fisher’s exact test was used for calculation of *p* values of categorical data.

*IST* intrasinus thrombolysis, *MRI* magnetic resonance imaging, *mRS* modified Rankin Scale, *MT* mechanical thrombectomy.

After 3–4 months, complete recanalization was achieved in 43 (52.4%) and partial one in 33 (40.2%) patients; recanalization was achieved in 93.4% of females and 90.5% of males. Complete recovery was achieved in 41 (50%), excellent clinical outcome (modified Rankin Scale [mRS] 0–1) in 67 (81.7%) and favorable clinical outcome (mRS 0–2) in 75 (91.5%) patients. Out of the remaining 7 (8.5%) patients, 2 (2.4%) patients died and 5 (6.1%) patients presented with unfavorable clinical outcome (mRS 3 in 3 and mRS 4 in 2 cases). The correlation between the initial stroke severity and clinical outcome after 3–4 months was not statistically significant (r = 0.3, p = 0.25).

Results of the multivariate logistic regression analysis identifying significant independent predictors of the observed parameters are shown in Table 3. Use of IST with rtPA (± MT) was associated with the presence of impaired consciousness and the presence of hemorrhage on initial magnetic resonance imaging (MRI). MT (± IST) was used more frequently also in the case of the presence of hemorrhage on initial MRI and in patients with thrombosis of the right transverse sinus. Complete recanalization was achieved less frequently in patients with thrombosis of deep veins. Pathological finding on control MRI (grades II–IV according to Kawaguchi et al.^5^) was associated with the presence of impaired consciousness and the presence of hemorrhage on initial MRI and occurred less frequently in patients with thrombosis of the left sigmoid sinus. The presence of focal neurologic deficit, thrombosis of the superior sagittal sinus and of the left transverse sinus were identified as significant independent negative predictors and, thrombosis of cortical veins as significant independent positive predictor of the achievement of complete recovery (mRS 0). In the case of excellent clinical outcome (mRS 0–1), female sex and the presence of focal neurologic deficit were identified as significant independent negative predictors and, the presence of GSRF as significant independent positive predictor.

**Table 3:** Significant independent predictors of the observed parameters in the whole set of 82 patients—results of multivariate logistic regression analysis.

Observed parameter	Predictor	OR (95% CI)	*p* value	Power *γ*
Use of IST (± MT)	Impaired consciousness	5.06 (1.38–31.25)	**0.0228**	**0.5307**
	Hemorrhage on initial MRI	6.85 (1.38–31.24)	**0.0180**	**0.6765**
Use of MT (± IST)	Hemorrhage on initial MRI	17.84 (3.52–90.4)	**0.0005**	**0.9357**
	Right transverse sinus thrombosis	92.55 (5.30–1662)	**0.0019**	**0.8702**
	Right sigmoid sinus thrombosis	0.055 (0.004–0.80)	**0.0326**	**0.5737**
Complete recanalization	Deep veins thrombosis	0.29 (0.098–0.86)	**0.0249**	**0.6080**
Pathological finding on control MRI	Impaired consciousness	8.12 (1.80–42.11)	**0.0072**	**0.7402**
	Hemorrhage on initial MRI	8.78 (2.26–33.66)	**0.0017**	**0.8835**
	Left sigmoid sinus thrombosis	0.24 (0.035–0.41)	**0.0368**	**0.6229**
Complete recovery (mRS 0)	Focal neurologic deficit	0.20 (0.065–0.56)	**0.0024**	**0.8338**
	Superior sagittal sinus thrombosis	0.21 (0.069–0.75)	**0.0148**	**0.7270**
	Left transverse sinus thrombosis	0.20 (0.062–0.63)	**0.0058**	**0.7766**
	Cortical veins thrombosis	3.85 (1.04–20.41)	**0.0435**	**0.4266**
Excellent clinical outcome (mRS 0–1)	Female sex	0.11 (0.018–0.70)	**0.0189**	**0.6567**
	GSRF	15.63 (3.03–83.33)	**0.0011**	**0.9017**
	Focal neurologic deficit	0.16 (0.033–0.71)	**0.0165**	**0.6483**

Values are expressed as OR (95% CI). Significant *p* values are indicated in bold.

*CI* confidence interval, *GSRF* gender-specific risk factors, *IST* intrasinus thrombolysis, *MRI* magnetic resonance imaging, *mRS* modified Rankin Scale, *MT* mechanical thrombectomy, *OR* odds ratio, *rtPA* recombinant tissue plasminogen activator.

## Discussion

Currently, conclusive evidence of the best treatment of CVST based on the results of randomized trials is missing. The standard care, i.e., anticoagulants administered in order to prevent further progression of the thrombus and thus also further deterioration of the clinical condition, are unable to dissolve the thrombus already occluding the sinus^6–8^. Partial or complete recanalization has been reported to occur in 47–100% when this therapy is used^9,10^. The recanalization process starts early—in the study performed by de Sousa et al*.*, recanalization was detected already on the 8th day in 74% of 68 patients (partial in 68% and complete in 6% of cases), and was not associated with sex, prothrombotic state and extent of thrombosis^11^. Recent guidelines prefer low-molecular-weight heparin in the acute phase^12^, however, therapy with unfractionated heparin is also being used^13^. In patients, who continue to deteriorate despite systemic anticoagulation therapy, endovascular therapy should be considered according to several guidelines and consensus statements^14–17^.

Historically, local pharmacological thrombolysis is the oldest method^18^. Currently, IST is performed by introducing a catheter via the femoral or internal jugular vein and rtPA is delivered directly to the site of the thrombus, while developing an effort to disrupt the clot to increase the surface area exposed to rtPA^19–22^. Studies using IST are listed in Table 4^23–31^. The problem of severity of hemorrhagic complications of IST has been discussed. Published literature reviews reported 9.8–10.7% of major hemorrhagic complications including intracranial bleeding, out of which 3.0–7.6% cases were symptomatic^32,33^. The presence of cerebral bleeding is not considered as a contraindication of anticoagulation or endovascular therapy^2,14,20,32,34,35^.

**Table 4:** Studies using intrasinus thrombolysis in the treatment of cerebral venous sinus thrombosis.

Authors	Number of patients	Type and dose of IST	Combination with MT	Outcome		Pre-treatment ICH	Worsening of ICH
				Recanalization	mRS		
Kumar et al*.* (2010)	19	Urokinase: bolus 100,000 IU, continued by 100,000 IU/h, max. 600,000 IU	None	Complete: 5 Partial: 7 No: 7	0–2: 15 3–4: 0 5–6: 4	6/19 (32%)	2/6 (33%)
Guo et al*.* (2012)	37	Urokinase: 1,000,000 IU daily, 5–7 days	None	Complete: 30 Partial: 5 No: 2	0–2: 34 3–4: 1 5–6: 2	23/37 (62%)	2/23 (9%)
Mohammadian et al*.* (2012)	26	rtPA: total 30 mg in 30 min	None	complete: 25 partial: 0 no: 1	0–2: 25 3–4: 1 5–6: 0	not available	
Li et al*.* (2013)	52	Urokinase: total from 100,000 to 1,500,000 IU (240,000 IU/h)	All	complete: 45 partial: 3 no: 4	0–2: 40 3–4: 6 5–6: 6	29/52 (56%)	4/29 (14%)
Garge et al*.* (2014)	10	Urokinase: bolus 100,000 IU, continued by 60,000 IU/h, 10–18 h	None	Complete or partial: 10 No: 0	0–2: 9 3–4: 0 5–6: 1	10/10 (100%)	1/10 (10%)
Zhen et al*.* (2015)	8	rtPA: 20 mg daily, 4–6 days	All	Complete: 5 Partial: 2 No: 1	0–2: 7 3–4: 0 5–6: 1	2/8 (25%)	0/2 (0%)
Karanam et al*.* (2016)	29	rtPA: bolus 2 mg, continued by 1 mg/h, 11–16 h	None	Complete or partial: 29 No: 0	0–2: 27 3–4: 1 5–6: 1	29/29 (100%)	0/29 (0%)
Mathukumalli et al*.* (2016)	24	Urokinase (8 patients): bolus 100,000 IU, continued by 70–80,000IU/h, max. 3 days rtPA (16 patients): bolus 10 mg, continued by 1 mg/h, max. 3 days	None	Complete: 4 Partial: 18 No: 2	0–2: 10 3–4: 8 5–6: 6	14/24 (58%)	0/14 (0%)
Guo et al*.* (2019)	156	Urokinase: 1,000,000 IU daily, 5–7 days	None	Complete: 112 Partial: 28 No: 16	0–2: 120 3–4: 25 5–6: 11	17/156 (11%)	3/17 (18%)

*ICH* intracerebral hemorrhage, *IST* intrasinus thrombolysis, *mRS* modified Rankin Scale, *MT* mechanical thrombectomy, *rtPA* recombinant tissue plasminogen activator.

In the 1990s, the MT started to be used in the treatment of CVST^36,37^. MT provides a significantly higher chance of successful recanalization, which is also rapid compared to IST alone, and is associated with a lower risk of bleeding complications when used without IST. Various devices (today, most frequently stent-retrievers) have been used with or without concurrent IST, while each has its specific limitations^8,20,38–40^. The fact that this therapy increases the chance of the achievement of a good outcome has been confirmed by the meta-analysis of 235 patients treated with MT for an unfavorable development of CVST. Concomitant IST was applied in 87.6% of patients; 40.2% of patients presented with symptoms of encephalopathy or were comatose at the beginning of the therapy. Despite that, 76% of patients achieved a good outcome (mRS 0–2) and, 14.3% died^41^. As shown by these results, aggressive therapy in severe CVST cases provided outcomes similar to those achieved in the non-selected group in the International Study on Cerebral Vein and Dural Sinus Thrombosis (ISCVT) where poor outcomes were observed in 13% of patients^3^. Recently published analysis of the set of 82 patients compared safety and efficacy of MT with intraoperative local thrombolysis versus MT with continuous thrombolysis for treatment of CVST. Near to complete recanalization was documented in 100% of patients (complete recanalization in 41%), 82% of patients achieved a good outcome and, 4% of patients developed postoperative intracerebral hemorrhage. No significant differences were found in the recanalization rate or prognosis between compared endovascular therapeutic methods^42^. The optimal treatment approach remains uncertain in the rare subgroup of CVST affecting the deep venous system, leading to more frequent venous infarctions and parenchymal bleeding. The prognosis of these patients is worse compared to other CVSTs with up to three times higher mortality^14^. The results of a recent meta-analysis of 120 patients from 69 studies, of which 40% were treated with IST and 20% with mechanical endovascular modalities, suggest that early endovascular treatment should also be considered in this subgroup of CVST. Despite the fact that a larger proportion of patients treated endovascularly had an initial impairment of consciousness (IST 47.5%, mechanical treatment 61.1% vs. anticoagulation 36.3%), mortality in particular treatment subgroups was similar (IST 17.0%, mechanical treatment 20.8%, anticoagulation alone 15.6%)^43^. The use of mechanical devices is limited in cases of cortical venous thrombosis, although the development of new, more flexible devices may decrease the risk of perforation also in these cases.

The recent European guidelines did not formulate a clear opinion on endovascular therapy of CVST; these guidelines only suggest avoiding this therapy in patients with a low probability of poor outcomes^12^. In the multicentre prospective TO-ACT trial, CVST patients with a high probability of poor outcomes (with the presence of one or more of the following factors: altered mental status, coma, thrombosis of the deep cerebral venous system, intracranial hemorrhagic lesion) were randomized to IST, mechanical thrombosuction, or a combination of both, compared with standard anticoagulation therapy^44^. The trial was stopped early for futility after enrolling 63 out of the planned 164 subjects (due to slow recruitment). No difference was found in rates of the primary endpoint between the endovascular versus standard treatment groups—a good outcome (mRS 0–2) was achieved at 12 months in 65 versus 66% of patients^45,46^.

In our patient set, we also opted for endovascular therapy in patients in a severe condition. The use of IST and of MT including their mutual combination was associated with the presence of hemorrhage on initial MRI, and the use of IST (± MT) was also associated with the presence of impaired consciousness. Endovascular therapy was used more frequently in women in whom impaired consciousness, focal deficit and thrombosis of the deep cerebral venous system were more frequently observed. MT (± IST) was used more frequently also in patients with thrombosis of the right transverse sinus. Thrombosis of the right transverse and sigmoid sinus is associated with elevated risk of parenchymal hemorrhage, as confirmed in the ISCVT study^3^, likely as a consequence of increased venous congestion due to common hypoplasia of the contralateral sinus.

Partial or complete recanalization was found in almost 93% of our patients after 3–4 months while no significant difference was found in the achieved recanalization between the sexes. Complete recanalization was achieved less frequently in patients with thrombosis of deep veins. By limiting venous drainage considerably due to the missing collaterals, thrombosis of the deep venous system is often a cause of bilateral venous infarctions in the basal ganglia and in the thalamus, commonly associated with impaired consciousness, and is considered as a predictor of a poor outcome^47–50^.

Hindered venous drainage due to CVST results in an edema and bleeding of brain tissue, found in about one third of patients in the acute phase. The situation was similar also in our set. The presence of pathological findings on control MRI in our patients was associated with the presence of impaired consciousness and the presence of hemorrhage on initial MRI and occurred less frequently in patients with thrombosis of the left sigmoid sinus. The lower probability of permanent tissue damage when the left sigmoid sinus is involved is apparently related to common hypoplasia of this sinus^49,51,52^ and, higher disposition of this sinus to early recanalization may be another explanation^11^.

The presence of focal neurologic deficit (such as hemiparesis, phatic disorders, alexia and agraphia), thrombosis of the superior sagittal sinus and of the left transverse sinus were identified as significant independent negative predictors and, thrombosis of cortical veins as significant independent positive predictor of the achievement of complete recovery (mRS 0). Coutinho et al.^53^ also noted a higher probability of complete recovery among 116 patients with an isolated involvement of cortical veins. Clinical presentation in the acute phase was less dramatic—the patients presented less frequently with the signs of intracranial hypertension, exhibited a longer interval from onset of the first symptoms to the diagnosis (7 days) and only 2% suffered from impaired consciousness. The diagnosis of an isolated cortical vein thrombosis is difficult and the pathology may be missed. We have no explanation why the thrombosis of the left transverse sinus has been identified as a significant independent negative predictor of complete recovery, given that the right transverse sinus is usually considered as such a predictor as confirmed by the ISCVT study, as well^3^. The left transverse sinus is often hypoplastic and the occlusion of a minor sinus is usually not as important.

In the case of excellent clinical outcome (mRS 0–1), female sex and the presence of focal neurologic deficit were identified as significant independent negative predictors and, the presence of GSRF as significant independent positive predictor in our study. The higher probability of achieving an excellent clinical outcome in women with GSRF may be related to their significantly younger age. More commonly, it is explained by their good health condition, because HC, the most common GSRF, is not prescribed to women with a higher risk who are also less likely to become pregnant^54,55^.

Several limitations of our study should be mentioned. First, its retrospective observational character, which might be associated with possible information bias. Second, some of the significant associations identified using the multivariate logistic regression analysis have wide confidence intervals. Third, the multiple comparison problems may increase type I error.

In conclusion, in our CVST group, both IST and MT were used more frequently in patients with the presence of hemorrhage on initial MRI. IST (± MT) was also more frequent in patients with impaired consciousness and, MT (± IST) more frequent in patients with thrombosis of the right transverse sinus. The presence of GSRF was identified as significant independent positive predictor and, female sex and the presence of focal neurologic deficit as significant independent negative predictors of excellent clinical outcome.

## Materials and methods

### Ethical approval and patients consent

The entire study was conducted in accordance with the Declaration of Helsinki of 1964 and its later amendments (including the last in 2013). All procedures were performed in accordance with institutional guidelines and regulations. The study was approved by the Ethics Committee of the University Hospital Hradec Králové (approval No. 202003 S17P). All conscious patients signed informed consent forms for the eligible and available treatment. Independent witnesses verified the signatures for cases in which there were technical problems. Due to the retrospective character of the study, the Ethics Committee did not require informed consent to include patients in the study.

### Patients and inclusion criteria

In a bi-centric retrospective study, performed using data from hospital registries (including both in-patient and outpatient medical records) of the Comprehensive Stroke Centers of the University Hospital Hradec Králové and University Hospital Ostrava, Czech Republic, the set consisted of all 82 consecutive CVST patients treated between January 2006 and March 2015 and meeting the inclusion criteria (availability of all observed clinical data and at least two comparable brain and venous system MRI scans—during the acute phase and after 3–4 months). There were no exclusion criteria. No patient was excluded due to lack of information.

### Observed parameters

The following demographic and clinical data were collected and evaluated: age, gender, presence of GSRFs (HC, pregnancy, puerperium, HRT) and other thrombophilic conditions, clinical presentation during the acute phase of the disease (including the presence of focal neurological deficit such as hemiparesis, phatic disorders, alexia and agraphia or focal epileptic seizures, the presence of impaired consciousness and, stroke severity evaluated by a certified neurologist using the National Institutes of Health Stroke Scale [NIHSS]), location and extent of venous sinus impairment, type of treatment (anticoagulant only, IST with administration of rtPA [Actilyse, Boehringer Ingelheim, Ingelheim am Rhein, Germany] and/or MT), recanalization, presence of parenchymal lesions and, clinical outcome after 3–4 months (evaluated during the follow-up visit by a certified neurologist and assessed using the mRS, with complete recovery defined as mRS 0 and excellent clinical outcome defined as mRS 0–1).

### Magnetic resonance imaging

Magnetic resonance imaging, using the same imaging protocol as in our previous study^55^, was performed using 1.5-T Magnetom Avanto and Magnetom Symphony apparatuses (Siemens, Erlangen, Germany).

In both scanners, the routine imaging protocol included T2 2D Turbo spin echo, 2D fluid-attenuated inversion recovery (FLAIR), 2D T2* gradient echo and diffusion weighted imaging (DWI) sequences with a b factor of 0, 500 or 1,000 and reconstructed apparent diffusion coefficient (ADC) maps, as well as a sagittal T1 spin echo sequence. Axial sequences were applied at an identical level using the same slice thickness (5 mm with a 20% gap) and slice number (25) using a rectangular field of view (FOV) of 230 × 190 mm. The bicallosal line was used as an anatomical landmark^55^.

For rapid visualization of the venous sinuses, we performed 2D phase contrast venography (slice thickness of 30 mm, FOV of 220 mm) at an encoding velocity of 20 cm/s in the sagittal and axial orientations, followed by 2D time of flight (TOF) venography performed routinely in all patients in the coronal plane (slice thickness of 3 mm with a 30% overlay, FOV of 200 mm, matrix of 192). 3D contrast magnetic resonance venography (3D volume-interpolated T1w fast low angle shot [FLASH] sequence at an isotropic resolution of 0.9 mm) was performed following the intravenous administration of a standard dose of the contrast agent gadolinium in inconclusive cases (e.g., hypoplastic dural sinuses and low flow areas, representing a major difficulty in 2D TOF)^55^.

A certified neuroradiologist blinded to the patient clinical data evaluated the MRI scans, assessing the location and severity of venous sinus impairment during the acute phase, presence of focal cerebral tissue lesions (edema and/or hemorrhage), and the extent of recanalization after 3–4 months. The morphological results were analyzed in combination with the results of venous magnetic resonance angiography and, including contrast administration in 5 cases to identify the anatomical variant (e.g., hypoplastic sinus vs. sinus occluded by a thrombus)^55^.

A modified scoring system developed by Zubkov et al*.* was used to quantify sinus impairment—with one point applied for each individual compromised sinus or cortical vein and two points applied for the impairment of the deep venous system^50^.

The changes in the brain tissue, as revealed by MRI, were graded as I–IV according to Kawaguchi et al*.*: grade I—tissue without structural changes, grade II—edema, grade III—edema and disruption of the blood–brain barrier, identified as opacification after contrast administration and, grade IV—apparent hemorrhage^5^.

### Statistical analysis

The normality of the distributions was confirmed using the Kolmogorov–Smirnov test. Fisher’s exact test was used to assess the relationship between the type of treatment, pathological findings on control MRI, complete recanalization, complete recovery (mRS 0) and excellent clinical outcome (mRS 0–1) and, observed categorical parameters. Unpaired two-tailed t-test was used, after confirmation of data normality, to evaluate the impact of quantitative parameters. Pearson correlation coefficient was used for the assessment of correlation between the initial stroke severity (assessed using the NIHSS) and clinical outcome after 3–4 months (assessed using the mRS). Standard multivariate logistic regression was separately used for each binary output variable of our interest. First, we examined possible hidden relationships between output variables and explanatory ones as they could cause difficulty to estimation process on one hand, but could also provide direct implications between these two groups of variables. Explanatory variables involved in such relationship with an output variable were excluded from the data set when estimating the corresponding model for this output variable. Then complete models were estimated using all input variables without hidden relationships with the dependent variable. As not all estimated coefficients in the complete models were significant at the level of 0.95, we examined the justification of their inclusion into the model with the likelihood ratio (LR) test. Due to the large number of explanatory insignificant variables and their possible mutual dependency in many cases, the testing was proceeded as follows. First, we removed all statistically insignificant variables from the complete model and used the LR test to verify whether such exclusion was statistically justifiable. If not, we put back the previously eliminated variables into the model in a piecewise mode according their *p* value in the full model until the null hypothesis of excluding the rest of them was not rejected by the LR test. For all explanatory variables included in final models we calculated their odds ratio, their corresponding confidence intervals, and the power of logistic regression. Detailed information on logistic regression analysis can be found in the paper by Vittinghoff et al*.*^56^. Statistical analysis was performed using a MatLab Statistics Toolbox version 2014b (MathWorks, Natick, MA, USA) and Eviews 8.0 (IHS Global Inc., Irvine, CA, USA) for verification.

## Data Availability

Data are available from the corresponding author on reasonable request.
